# A Case Report on Dysgraphia in a Patient Receiving Blinatumomab: Complex Characters Are Easy to Find in a Handwriting Test

**DOI:** 10.3390/medicina58060733

**Published:** 2022-05-29

**Authors:** Yasuto Yamamoto, Takeo Shimasaki, Yasuhito Ishigaki, Shino Fujimoto, Yoshimitsu Takahashi, Shiori Kimura, Keiko Aijo, Mami Takayanagi, Shuichi Mizuta, Togen Masauji, Yasufumi Masaki

**Affiliations:** 1 Department of Pharmacy, Kanazawa Medical University Hospital, 1-1 Daigaku, Uchinada, Kahoku 920-0293, Japan; yamar@kanazawa-med.ac.jp (Y.Y.); takappe@kanazawa-med.ac.jp (Y.T.); masauji@kanazawa-med.ac.jp (T.M.); 2 Medical Research Institute, Kanazawa Medical University, 1-1 Daigaku, Uchinada, Kahoku 920-0293, Japan; ishigaki@kanazawa-med.ac.jp; 3 Tottori Prefectural Central Hospital, 730 Ezu, Tottori 680-0901, Japan; shino_44_store@yahoo.co.jp; 4 Department of Nursing, Kanazawa Medical University Hospital, 1-1 Daigaku, Uchinada, Kahoku 920-0293, Japan; yabu@kanazawa-med.ac.jp (S.K.); k1608@kanazawa-med.ac.jp (K.A.); 5 Department of Nursing, Kanazawa Medical University Himi Municipal Hospital, 1130 Kurakawa, Himi 935-0025, Japan; taka-m@kanazawa-med.ac.jp; 6 Department of Hematology and Immunology, Kanazawa Medical University, 1-1 Daigaku, Uchinada, Kahoku 920-0293, Japan; mizuta@spacelan.ne.jp (S.M.); yasum1macky1@gmail.com (Y.M.)

**Keywords:** blinatumomab, handwriting test, adverse effects, dysgraphia

## Abstract

Recent advances in chemotherapy have led to the emergence of new types of anticancer agents. With these advances, cases of side effects that have not been witnessed in the past have emerged. The systems of side effect evaluation and their grading have been based on the existing knowledge, such as the CTCAE (Common Terminology Standard for Adverse Events) for evaluating adverse drug reactions in cancer chemotherapy clinical trials. Therefore, new types of side effects may be overlooked or underestimated. Blinatumomab is a bispecific T-cell–engager (BiTE) antibody with specificity for CD19 on B cells and CD3 on T cells. Neurological events, such as neuropathy and encephalopathy, are serious side effects of BiTE antibodies. We encountered a case of a 62-year-old woman who experienced short-term memory impairment and dysgraphia after the first blinatumomab administration for Philadelphia chromosome negative (Ph−) B-cell acute lymphoblastic leukemia (ALL). The CTCAE does not include dysgraphia as a classifier for antibody therapies, such as blinatumomab, and immune effector cell-associated neurotoxicity syndrome, which is defined as a Chimeric antigen receptor T cell therapy-related toxicity; dysgraphia is included in the list of symptoms but is not graded. In this case, the severity of dysgraphia differed depending on the complexity of the letters examined. There is no report that the severity of dysgraphia depends on the letters’ complexity, and therefore, it may be overlooked when using simple letters. We have reported the characteristics of dysgraphia in this case and the differences observed when judging different letters.

## 1. Introduction

Although more than 80% of patients with acute lymphoblastic leukemia (ALL) achieve hematological remission following multidrug therapy, more than 50% experience disease recurrence and fewer than 20% exhibit resistance to initial treatment [1]. Despite the use of multiple salvage therapies, the remission rate with these treatment regimens remains low. Hence, long-term survival is low among the patients [2,3].

Novel antitumor agents have emerged in recent years [4,5]. Blinatumomab was the first Bispecific T-cell-engager (BiTE) antibody with specificity for both CD19 expressed on B cells and CD3 expressed on T cells [6]. This immunotherapeutic drug exerts antitumor effects by activating T cells via cross-linking B-cell ALL (B-ALL) cells and T cells [6,7,8,9,10]. In multiple international clinical trials of blinatumomab combined with existing standard chemotherapies in patients with relapsed or refractory B-ALL, blinatumomab was associated with a significantly higher rate of complete hematological remission and longer overall survival [5,11,12].

As mentioned above, new types of anticancer agents have been developed. With these advances, side effects that have not been witnessed earlier have emerged in some cases, but the evaluation and grading of side effects have been conducted under a system based on previous findings. Hence, new types of side effects may be overlooked or underestimated.

In clinical trials of blinatumomab, many patients experienced adverse events [5,11,12]. The major ones include cytokine release syndrome, fever, neutropenia, thrombocytopenia, elevated liver enzymes, back pain, and headache. Of these, neurotoxicity, including encephalopathy, seizures, confusion, and aphasia, is the most serious adverse event and is among the most common reasons for treatment discontinuation [13,14].

The International Council for Harmonisation of Technical Requirements for Pharmaceuticals for Human Use (ICH) [15] guidelines established in 1998 recommended the use of common definitions and evaluation criteria to promote common understanding, evaluation, and acceptance of clinical trial data from foreign countries. Currently, the Common Terminology Standard for Adverse Events(CTCAE) [16] is widely used as the standard for evaluating adverse drug reactions in cancer chemotherapy clinical trials.

The CTCAE was established by the National Cancer Institute. The general grades are classified into five levels. Moreover, the details of the grade classification are defined for each adverse event. Owing to these efforts, most adverse reactions are correctly assessed. However, as mentioned above, new types of adverse reactions due to new types of anticancer agents may not be included in the CTCAE guidelines or may not be graded. The latest CTCAE has been updated and allows for better and faster evaluation of neurologic toxicity due to antibody therapy, but there is no grading of dysgraphia.

The American Society for Transplantation and Cellular Therapy (ASTCT) established guidelines for the evaluation of side effects related to immune effector cell-associated cytokine release syndrome because the CTCAE guidelines were insufficient. The ASTCT guidelines define immune effector cell-associated neurotoxicity syndrome and list dysgraphia as one of the symptoms, but no actual grading has been made [17].

As a neurological side effect, dysgraphia is also cited in blinatumomab’s guidelines and a writing test is recommended [18]. However, it is unclear which letters are best suited for assessing the presence and extent of neurological damage at an early stage. Additionally, the frequency of neurological damage is unclear, and detailed reports are rare.

We experienced a case of dysgraphia after chemotherapy with blinatumomab. Dysgraphia, sometimes termed agraphia, is a specific deficiency in the ability to write, which is not associated with a deficiency in the ability to read or an intellectual impairment. In this case, the patient does not have a reading disorder or intellectual impairment.

There have been few reports of chemotherapy-induced dysgraphia or agraphia, with the only report of dysgraphia being associated with neurologic toxicity in chimeric antigen receptor T-cell therapy [19].

Based on a case in which the writing disability was applicable to complex letters only, we believe that writing disorders may be easily missed when using simple letters. Therefore, we have reported the details of a case of dysgraphia caused by blinatumomab.

## 2. Case Presentation

A 62-year-old woman was diagnosed with Ph-negative pre-B-ALL 4 years prior to presentation. She was treated according to the Japan Adult Leukemia Study Group ALL-202 protocol [20]. Treatment was discontinued because of severe allergic reactions to l-asparagine and a syndrome of inappropriate antidiuretic hormone secretion caused by vincristine. Subsequently, the patient received four courses of hyper-CVAD therapy. She experienced an isolated bone marrow relapse 2 years after completing the therapy and achieved hematological remission after three courses of inotuzumab ozogamicin. The patient was then admitted to the hospital to receive blinatumomab as consolidation therapy. Her consciousness was clear, and she exhibited no neurological deficits, including memory impairment and dysgraphia. She could speak with medical staff and sign documents by herself at the time of admission. Her medical history included Graves’ disease, but she was not on any medication then. She did not have Parkinson’s disease or hand tremors that would affect her writing. Her family history also showed no neurological, psychiatric, or developmental disorders.

### 2.1. Methods

**Figure 1:**
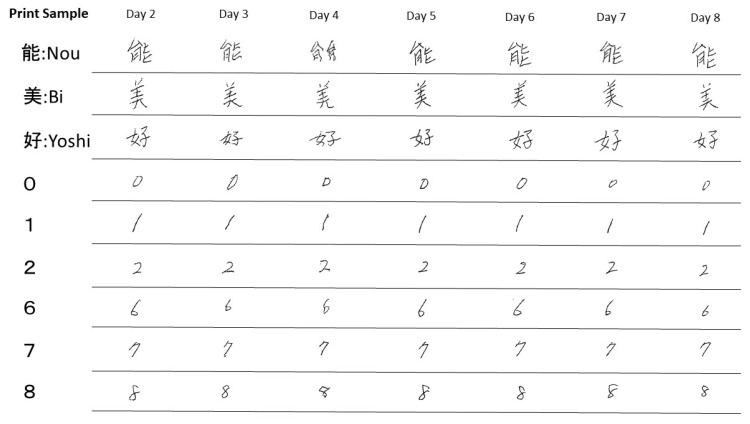
Characteristic changes in the patient’s written characters (partially extracted). It was easier to identify changes in Kanji characters than those in numbers. One Japanese character has multiple meanings. For example, “Nou” is the power to do work, work, action, effect, efficacy, working, technique, place name, etc. The character for “Bi” means fine or beautiful in appearance, excellent or good in content, or praiseworthy. The character for “Yoshi” means loving, preferring, beautiful, good looking, good, just right. Combinations of several letters indicate a particular meaning. For example, combining “Bi” with “Kei”, meaning “shape” means especially good looking.

After commencing blinatumomab administration, the nurses and pharmacists carefully monitored the patient for adverse events. A descriptive test was administered for the early detection of neurotoxicity during blinatumomab administration. The characters used in the test are presented in Figure 1. Kanji is a writing system that originated in the ancient Chinese civilization of the Yellow River, but it is currently used to write the Chinese, Japanese, Korean, and Vietnamese languages. In this study, “Kanji” indicates the independently developed Chinese characters used in Japan.

The nurses and pharmacists evaluated the depicted writing styles as well as the waviness and balance of the letters. Specifically, the numbers “0, 1, 2, 6, 7, and 8” and the Kanji characters “Nou, Bi, and Yoshi” were evaluated because these are characters that the patients typically draw on a daily basis. In case of identifying an abnormality, it was reported to the doctor.

### 2.2. Progress after Hospitalization

On Day 3 of blinatumomab administration, a hand tremor appeared but the written numbers and Kanji characters were legible. The patient was judged to not have dysgraphia. On Day 4, “Nou” was wavy and illegible, and thus, the patient was considered to have a writing disorder by several medical personnel (Figure 1).

**Figure 2:**
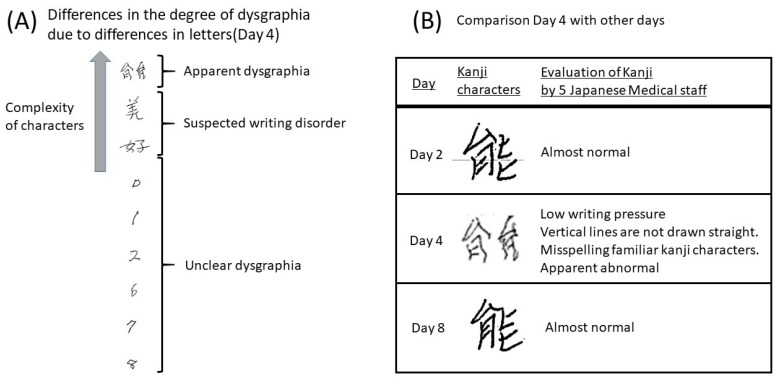
Characteristics of dysgraphia. (**A**) Differences in the severity of dysgraphia attributable to differences in letters. In case of complex characters, dysgraphia was evident. (**B**) Comparison with other days for letters for which dysgraphia was detected on Day 4. Based on the judgment of five medical personnel, the writing on Day 4 was deemed abnormal.

As presented in Figure 2, the patient’s writing pressure was reduced, vertical lines could not be drawn, and some of the letter components were clearly wrong. These findings suggested a central nervous system disorder. Disturbance of consciousness was not noted, but clinical findings of aphasia and apraxia were observed and considered grade 3 neurological events.

**Figure 3:**
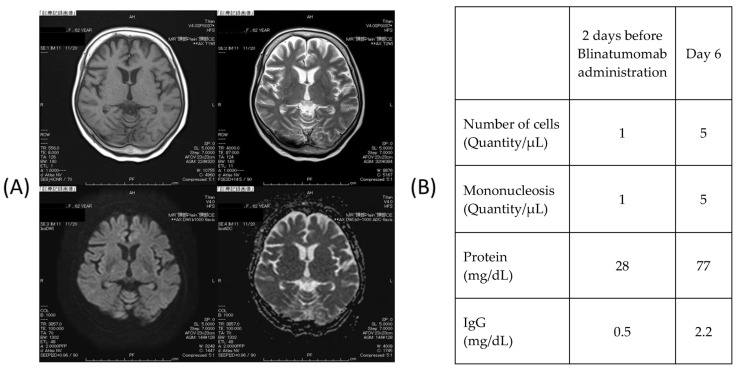
(**A**) Head magnetic resonance imaging. Findings: There is no obvious abnormal signal area in the brain parenchyma. There was no abnormal contrast area in the Gd. The pituitary gland was normal in size. The high T1WI signal in the posterior lobe was also maintained. (**B**) Cerebrospinal fluid test. The spinal fluid examination showed an increase in cerebrospinal fluid protein, so it is possible that some kind of encephalitis or meningitis had occurred, although it was not diagnosed.

After the onset of dysgraphia, brain magnetic resonance imaging (MRI) and a cerebrospinal fluid (CSF) test were performed but no obvious abnormality was detected. The CSF examination uncovered an increase in CSF protein levels, which indicated the possible development of encephalitis or meningitis despite no formal diagnosis (Figure 3).

A blood test administered 2 days after the appearance of dysgraphia did not reveal any specific changes (Table 1).

The patient was not taking any other medications that could have caused the neurological damage (Table 2).

**Figure 4:**
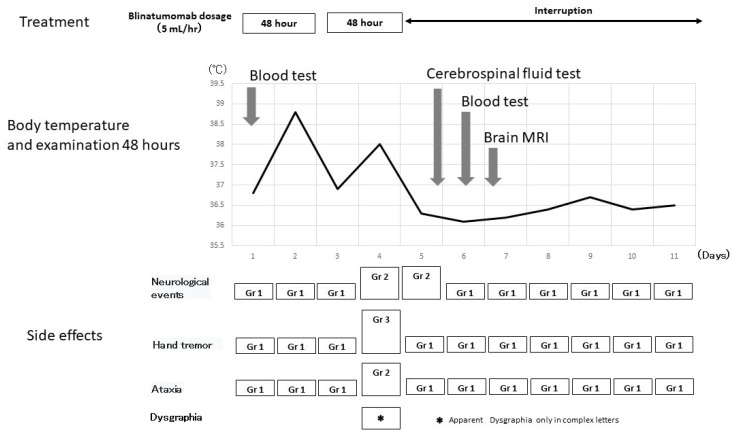
The clinical course of blinatumomab and side effects. Neuropathy appeared on Days 4–5. Brain magnetic resonance imaging and a cerebrospinal fluid examination did not reveal the cause of neuropathy. The patient’s symptoms improved rapidly after blinatumomab discontinuation. A writing disorder appeared on Day 4 of treatment. The severity of dysgraphia could not be graded because no grading system exists.

After the discontinuation of blinatumomab on Day 5, the character for “Nou” improved to legible, although a slight hand tremor remained. Vital signs and the level of consciousness were normal, and the patient’s written keywords were smooth. Because the onset of tremor and ataxia coincided with the beginning of dysgraphia and improved quickly after discontinuing blinatumomab, dysgraphia was considered a side effect of blinatumomab (Figure 4).

On day 12, >7 days after blinatumomab discontinuation, the adverse effects were sufficiently decreased to allow the second cycle of drug administration to be commenced on the following day.

## 3. Discussion

New chemotherapeutic agents, such as molecular targeted agents and antibody therapeutics, have been developed in recent years. The indications and measures for side effects have consequently become more complex [17,21]. In the past, cancer chemotherapy was generally administered in an inpatient setting so that if a problematic side effect occurred, appropriate action could be taken immediately. In recent years, chemotherapy has often been administered on an outpatient basis. Hence, infrequent side effects unnoticed by the patient are more likely to go undetected.

Blinatumomab is a BiTE antibody. Neurological events, such as cerebral neuropathy and encephalopathy, are the known serious adverse effects of this drug [5,11,12]. Although dysgraphia is mentioned in the guidelines, its frequency is unclear and detailed reporting has been rare. Dysgraphia is one of the most easily missed side effects because it cannot be detected without a writing test. When dysgraphia is present only in complex characters, it is rarely noticed, as in this case. Therefore, the fact that there have been no reports of side effects does not mean that there are no side effects. The use of a writing test to monitor the presence of dysgraphia was recommended in the NCCN guideline and a previous paper [18]. However, there is no mention of the type of writing test or the complexity of the writing that should be examined. Moreover, the CTCAE guideline does not classify the grade of dysgraphia. In a phase III study of blinatumomab, the rate of dysgraphia was 1.1% [5], but which specific failures occurred is unclear. There have been no reports on differences in the occurrence of dysgraphia depending on the type or complexity of the letters written on the same day or at the same time during blinatumomab administration. We speculate that dysgraphia is more likely to have been overlooked than to be rare. There were no evident abnormalities in the brain MRI and CSF examination data in this study.

It is important to differentiate the dysgraphia in this case from peripheral neuropathies, such as tremors. In the present case, the possibility of peripheral neuropathy is low because simple writing is not impaired in this case. In addition, the CSF examination showed mild abnormalities, and the fact that only complex letters were impaired suggests that a portion of the brain function might have been impaired. In fact, the concomitant temporary short-term memory impairment also implies that the writing disorder was caused by a central dysfunction.

The possibility of writing impairment due to drugs other than blinatumomab or other diseases is eliminated because we stopped blinatumomab immediately after the appearance of dysgraphia, and then the condition improved quickly. The patient had no current history of neuropathy. No drugs were administered before or after blinatumomab administration that could have caused major neuropathy, and concomitant medications while the patient was receiving blinatumomab had a minimal frequency of side effects of tremor and neuropathy. Meningitis and encephalitis were not detected. However, dysgraphia was detected only for complex letters, which alludes that this condition is not a symptom of peripheral neuropathy, but a sign of brain damage caused by the drug. In this case, all written letters were confirmed to be normal on Day 2 after chemotherapy. Meanwhile, numbers and the Kanji characters “Bi” and “Yoshi” were legible on day 4 but “Nou” was illegible, which was considered indicative of a writing disorder.

**Figure 5:**
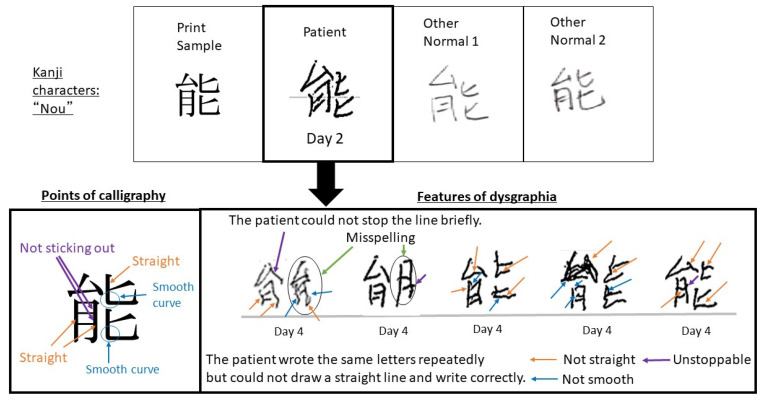
Features of dysgraphia. The patient could not stop the line briefly and draw a straight line. When displaying the abnormality, she could not repeatedly write Kanji characters. Failing to write these familiar Kanji characters is synonymous with failing to write one’s name. Some parts of the Kanji are wrong, which suggests a central nervous system disorder. In addition, the patient may have difficulties with fine motor skills.

On Day 5, 1 day after discontinuing blinatumomab therapy, the writing of “Nou” became legible. Blinatumomab has a short half-life of approximately 3 h. Thus, the rapid improvement of dysgraphia after blinatumomab discontinuation supports the conclusion that it was a drug-induced event. “Nou,” for which dysgraphia appeared, is composed of vertical, horizontal, and diagonal lines. The calligraphy on Day 2 did not reveal any abnormalities compared with other people’s calligraphy (Figure 5).

Blinatumomab-induced dysgraphia may be overlooked depending on the type of letters being written. The writing test is easier to evaluate when selecting letters with large changes at the time of the appearance of dysgraphia. This change could facilitate the early detection of dysgraphia and prevent it from being overlooked.

**Figure 6:**
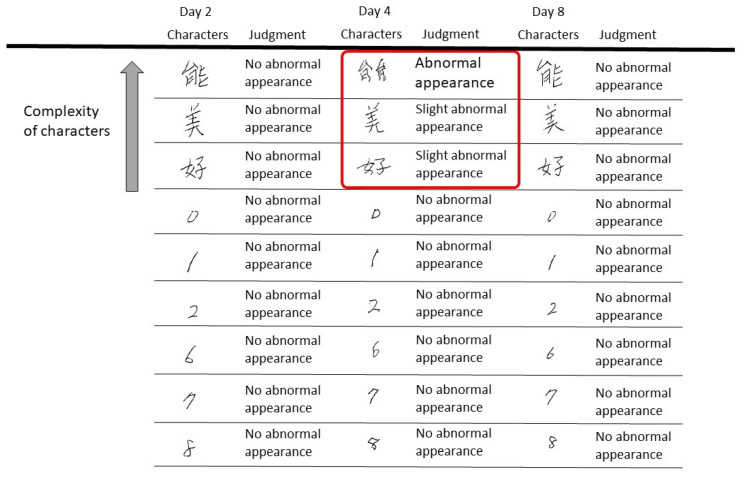
Evaluation of dysgraphia based on the complexity of characters. For numbers comprising relatively simple lines or circles, the severity of dysgraphia was low. It is not easy to determine whether the writing disorder occurred on the fourth day. Kanji characters with complex lines are more likely to show dysgraphia.

This case suggests that blinatumomab-induced neuropathy may be more easily detected using complex letters and that future writing tests should consider the complexity of the letters (Figure 6).

Furthermore, there is currently no grading system for determining the side effects of dysgraphia, and it is necessary to devise a system for grading such side effects as implemented for the side effects of other drugs. On Day 4, the patient was able to write simple letters but could not write the complex letter “Nou” with straight lines, which resulted in a misspelling that made the letter unrecognizable. We believe that a grading system for dysgraphia could potentially allow the early detection of the side effects of blinatumomab.

## 4. Conclusions

The appearance of dysgraphia in patients treated with blinatumomab may differ depending on the complexity of the letters. Performing a spelling test using complex letters may permit the early detection of the condition. Chemotherapy-induced dysgraphia may be overlooked because of the lack of established assessment methods. We hope that writing tests with more complex characters will be used worldwide and that future case accumulation will lead to additional grading of dysgraphia when it is evident that dysgraphia is being missed.

## Figures and Tables

**Table 1:** A blood test was conducted 2 days after the appearance of dysgraphia. The blood tests revealed no obvious changes.

Item	Reference Ranges	Day 6	Item	Reference Ranges	Day 6
WBC	3300–8600	2150/μL	TP	6.6–8.1	5.3 g/dL
Neut.	40–77	59.1%	Alb	4.1–5.1	3.5 g/dL
Lym.	16–44	30.1%	T-Bil	0.4–1.5	0.5 mg/dL
Eos.	1–7	1.6%	AST	13–30	20 U/L
Baso.	0–1	0.1%	ALT	7–23	20 U/L
Mono.	4–9	8.1%	LDH	124–222	NA
Blast.		NA	ALP	38–113	NA
RBC	3.86–4.92 × 10^6^	4.31 × 10^6^/μL	γ-GTP	9–32	39 U/L
Hb	11.6–14.8	13.2 g/dL	BUN	8–20	26 mg/dL
Hct	35.1–44.4	40.8%	Cre	0.46–0.79	0.45 mg/dL
MCV	83.6–98.2	94.6 fL	UA	2.6–5.5	3.0 mg/dL
Ret	0.8–2.1	1.3%	Na	138–145	142 mmol/L
PLT	15.8–34.8 × 10^4^	10.4 × 10^4^/μL	K	3.6–4.8	3.7 mmol/L
			Cl	101–108	103 mmol/L
			Ca	8.8–10.1	8.8 mg/dL
			CRP	0.00–0.14	0.03 mg/dL

**Table 2:** Drugs used during Blinatumomab administration. The patient was not taking any medications that could have caused the writing problems.

Drugs	Content and Frequency of Side Effects Associated with Peripheral Neuropathy (Drug Package Insert)	Prescription Intent
Dexamethasone sodium phosphate	No description	Prevention and Treatment of side effects
Hydrocortisone Sodium Succinate	Myopathy (Frequency unknown)	Treatment of side effects
Methylprednisolone Sodium Succinate	Myopathy (Frequency unknown) convulsions (Frequency unknown)	Treatment of side effects
Betamethasone sodium phosphate	No description	Treatment of side effects
Acetaminophen	No description	Clothes fever
Fluconazole	tremor (Frequency unknown)	Regular oral administration
Rabeprazole Sodium	Numbness in the limbs (Less than 0.1%) Weakness limbs (Less than 0.1%) Weakness (Less than 0.1%)	Regular oral administration
Enterococcus feacium Clostridium butyricum Bacillus subtilis	No description	Regular oral administration
Sulfamethoxazole trimethoprim	Numbness in the limbs (Less than 0.1%) Shivering limbs (Frequency unknown) Weakness (Frequency unknown)	Regular oral administration

## Data Availability

Data are available from the corresponding author upon reasonable request.
